# Situation Awareness Measurement in Remotely Controlled Cars

**DOI:** 10.3389/fpsyg.2021.592930

**Published:** 2021-04-20

**Authors:** Václav Linkov, Marek Vanžura

**Affiliations:** Autonomous Driving Department, CDV – Transport Research Centre, Brno, Czechia

**Keywords:** remotely controlled road vehicles, measurement, traffic safety, human factor, situation awareness, remotely controlled cars, remotely operated cars

## Abstract

This study reviews the current information concerning the measurement of the situation awareness (SA) of the teleoperated drivers of remotely controlled cars. The teleoperated drivers who drive these cars are in a remote location, and they control the cars through a communication interface. The objective methods with probes are beneficial in measuring SA on a closed circuit without real traffic. Questions specifically should address the information provided on the road by haptic sensations, such as the slope of the road and the vehicle's speed. Methods for measuring SA that involve probes and interruptions obviously are not suitable for use on public roads. A stable environment for the display and control of the communication interface is suitable for an eye tracker in measuring SA. These features also facilitate the use of subjective observer-rating methods. Both of these methods are suitable for driving on real roads because they are not intrusive. SA research in a real-road environment also should demonstrate how the SA of other drivers is affected by seeing a car without a driver. Given the remote character of driving, cultural differences in cognition may have a significant influence on the SA of the teleoperated driver.

## Introduction

Technological development allows for the operation of vehicles without a driver in the vehicle. Especially, the importance of autonomous vehicles has increased significantly, and it is likely to continue to do so (Alawadhi et al., 2020). However, even though fully autonomous cars currently are being developed, there will be situations in which automation must be supplemented by a person's involvement (Hampshire et al., 2020). For example, the automated controllers and devices may suddenly malfunction, leaving the autonomous car stranded. In such cases, as the passenger may not possess the driving skills to drive the car, a human teleoperator in an office can take control and operate the car remotely using video information and other data transmitted from the car through a wireless network (Berman, 2019). Besides, such remote operation can be useful in other situations, e.g., when the driver gets drunk and unable to control the vehicle. In such a case, the remote operator must have good driving skills to get the driver home safely. Therefore, it is important to determine how to assess the quality of people's remote driving skills. Several factors are crucial to the performance of people in unmanned systems, these include situational awareness (SA), cognitive workload, complacency, and overtrust of automated systems (Stark et al., 2012).

A remotely controlled vehicle (RCV) is a vehicle that is controlled by a person, i.e., a “teleoperator,” from a remote location using a communication interface. The vehicle contains a sensor module that provides information about the environment and other information to the teleoperator. Typical sensors are cameras, lidar systems, radars, and sonar. The teleoperator drives the vehicle from a remote control station. An RCV could be a train, an agricultural machine, an underwater vehicle (Ho et al., 2011), a drone (Chen et al., 2018), or a robot for destroying mines (Riley et al., 2004).

Remotely controlled cars (RCCs) are cars without drivers operated on public roads and driven by an operator in a remote location. Therefore, all RCCs are also RCVs. Currently, there is a lack of research regarding the SA of the RCCs operators. The RCCs operators are also referred to as “teleoperated drivers.” In this study, we provide directions concerning how to measure the SA of the RCCs' teleoperated drivers to assess their driving skills.

The rest of the paper is organized as follows: We review the concept of SA and its measurement in the next section. The following section analyzes the measurement of SA in the context of RCVs. The final section suggests an optimum approach for measuring SA as it relates to RCCs.

## Situation Awareness and its Measurement

Situation awareness is the ability to see and understand (Endsley, 2008), including what “that information means to you now and in the future” (Endsley and Jones, 2011:13). Gawron (2019:135) defines SA as “knowledge relevant to the task being performed.” SA usually is used in a specific context, and it is defined more specifically for that particular context (Endsley and Jones, 2011:13). SA can be defined for humans, but it also can be defined for self-directed systems that use artificial intelligence (Adams, 2007), such as autonomous vehicles (Freedman and Adams, 2007). SA for robots concerns the need to understand orders from people, to know how to act after understanding the orders, and collect information about other robots and people as part of their repertoire (Drury et al., 2006). SA is dependent on many factors and environmental characteristics. Table 1 summarizes driver, environment, and car characteristics that influence a driver's SA. Table 2 summarizes operator, environment, and operation interface characteristics that influence a teleoperator's SA.

**Table 1:** Driver, environment, and car characteristics that influence a driver's situational awareness (SA).

**Driver's characteristics**	
Age	SA is higher for experienced, middle-aged drivers. Younger and older drivers have lower SA values. Middle-aged drivers focus on other vehicles on the road, while older drivers focus on hazards associated with the road environment (Scott-Parker et al., 2020)
Cognitive abilities	SA is related to a driver's divided and selective attention ability (Chaparro et al., 1999). Divided attention and working memory capacity were found as increasing pilots' SA also by Cak et al. (2020). Working memory capacity, visual processing ability, time-sharing skills, and spatial perceptual ability were found to increase SA and fatigue to decrease SA by Yang et al. (2020). SA could be improved by training of metacognitive strategy—skills utilized while learning a new task (Soliman and Matha, 2009)
Driver's experience	When the screen goes black during the SAGAT, experienced drivers answer more correctly than inexperienced drivers. The differences between the two groups disappear if the last image on the screen is preserved (Jackson et al., 2009). Differences in eye fixation between novice and experienced drivers are found by the eye tracker. Tracking one's eyes influences driver's activity less than pauses and asking questions during SAGAT. The validity of using an eye tracker for the measurement of SA is supported by the fact that an operator's SA is related to the operator's fixation of her or his eyes on SA-relevant objects (Moore and Gugerty, 2010). In the context of driving a car, novice drivers fixate on lower positions, and they do so for a longer time than experienced drivers (Chapman and Underwood, 1998)
Driver's understanding of car operation	SA depends on providing the driver the correct description of the car's driving system (Blömacher et al., 2018)
Distraction	Distracted people are more risky drivers, e.g., they drive at higher speeds and have shorter headway distances (Yanko and Spalek, 2014)
Anger	SA deteriorates when a driver is angry (Jeon et al., 2014)
Mindfulness	Mindfulness increases SA, so it could be enhanced by mindfulness training (Kass et al., 2011)
**Environment characteristics**	
Variability of driving task	The operator's ability to focus her or his attention on driving deteriorates when the task is monotonous (Greenlee et al., 2018), e.g., when driving on a highway with few other cars
Conversation/phone call	SA is decreased by conversation (Heenan et al., 2014) or a phone call (Ma and Kaber, 2005; Ma et al., 2005; Kass et al., 2007)
Text messages	Hearing audio notification announcing the reception of a text message from a cell phone decreases SA for the next 10–30 s (Van Dam et al., 2020)
Previous events	SA increases in driving after the driver has seen or been involved in a hazardous event (Kaber et al., 2016)
**Car characteristics**	
Level of automation	SA was found to decrease as the level of automation increases in RCVs (Kaber et al., 2000; Onnasch et al., 2014) because the operator does not have to pay attention to the environment. Nevertheless, automatized driving could increase the driver's SA by releasing their attention capacity (Deng et al., 2020), e.g., the use of adaptive cruise control improves SA under normal driving conditions (Ma and Kaber, 2005; Ma et al., 2005). These seemingly contradictory results were explained by Bashiri and Mann (2014) in the research on drivers of semiautonomous vehicles: the negative relationship between the level of automation and the driver's SA exists only for activities the driver normally does not do automatically. If the steering wheel was autonomous, it did not influence the driver's SA because using a steering wheel did not require a lot of attention
Availability of taking control during operation	For autonomous car, SA is important during the takeover of operator's control (van den Beukel and van der Voort, 2017a) if it is possible to take control during the car's operation

**Table 2:** Remotely controlled vehicle (RCV) operator, environment, and operation interface characteristics that influence a teleoperator's situational awareness (SA).

**Operator's characteristics**	
Trust	The operator should trust the teleoperation system for better performance (Hancock et al., 2011). However, SA decreases as the trust in automation increases because of loss of attention (Endsley, 2017b)
Distraction	A remote driving task could be monotonous, so the teleoperator's mind may not be focused on driving, which reduces SA (Endsley, 2017a)
Computer games experience	RCVs have similar environments to those found in computer games. People with extensive experience in playing computer games in which the player has to develop good orientation in a 3D virtual environment might have better remote driving skills (Wheatcroft et al., 2017). Therefore, the SA of people who play these games might be higher. For example, Cuevas and Aguiar (2017) found that people who play first-person shooter games have a better spatial orientation when piloting remotely controlled drones
Cultural differences	It is hypothesized that SA differs in various cultural environments, e.g., Western cultures with analytical thinking and Eastern cultures with holistic thinking (Zhang et al., 2018). Cultural differences in cognitive style (e.g., holistic thinking, analytic thinking) might create differences among teleoperators
**Environment characteristics**	
Multitasking	Performing secondary tasks and multitasking reduces SA in the operation of an RCV (Ratwani et al., 2010)
Quality of signal transmission	SA in RCVs could be influenced by the poor quality of signal transmission caused by weather conditions (Porathe et al., 2014), the loss of connection, or the failure of a sensor (Riley et al., 2006)
**Operation interface characteristics**	
Number of cameras/available views	Operators recognize objects at a larger distance if more cameras are available (Ruddle et al., 1999); thus, having more available views increases the values of operators' SA (McDermott et al., 2005)
Simultaneous operation of more RCVs	Parallel operation of more RCVs might decrease SA (Riley et al., 2008), but Riley and Strater (2006) did not find a significant decrease
The complexity of the operational interface	SA could be enhanced if the engineers who design the operator interface would acknowledge that people have a limited capacity for memory and attention, and this capacity is diminished further by stress, noise, and vibrations. The interface should not be overly complex, and it should highlight only important data that would not overload the operator (Endsley and Jones, 2011:31; Papadimitriou et al., 2020)
Earcons/icons	SA increases if appropriate earcons (auditory cues) and icons are integrated into the operator's interface (Kaber et al., 2006)
Displays with additional information	SA is increased when there is a display that shows the positions and the predicted movements of objects near the RCV (Wiegand et al., 2019). SA also is supported if “chat with SA-relevant information” is added to the visual teleoperation interface (Robb et al., 2018)
Active vehicle approach	A car system's initiation of communication with the operator was found to increase SA when the driver was inactive for long periods (Lee et al., 2019). Human aid via a cell phone or an automated aid presented on a display improves SA for strategic behaviors, such as navigation (Ma and Kaber, 2006)
Sensations of the vestibular organs	In RCVs, SA could be reduced by sensations not felt by the vestibular organs and mechanoreceptors in the skin. This might be partially provided by systems to provide vestibular or somatosensory feedback, which support the operator's SA about the motion of RCVs (Pazuchanics et al., 2010). Cars with higher feedback might allow the driver to create better SA, but the difference found by Walker et al. (2008) was not significant
Operation interface awareness of operator's SA	SA could be supported using a system that identifies visual cues of a decline in the driver's SA, such as the position of the pupil of the eye (Hijaz et al., 2019)
Camera's ability to adjust to changes in the lighting	Cameras should be able to adjust during changes between open and enclosed spaces (Riley et al., 2006)

SA can be viewed from three perspectives (Salmon et al., 2012). The individual perspective is a purely cognitive phenomenon that exists in people's minds. The engineering perspective arises from the interaction between people, the displayed information, and other indicators. The system perspective is based on the interaction between people and various technologies, including displays and indicators. An example is a railway crossing, which includes the SA of the car driver, the train, and the train operator, and the railway crossing (Salmon et al., 2012). SA for emergencies is different from SA for behavior in normal situations (Wickens, 2000) because emergencies require the operator to pay more attention to the environment (Wu et al., 2019).

Many methods to measure SA range from self-rating scales to eye trackers (Zhou et al., 2019). SA measurement methods can be classified into three groups (Pew, 2008). The first group consists of direct performance methods that measure the results of the operator, and it is expected that these results are related to the operator's SA. Second, experimental methods that include queries, probes, and other techniques to measure the operator's behavior and SA knowledge during the performance. Third, SA could be measured by subjective methods based on either an observer's ratings or the operator's self-ratings. Subjective methods for the SA measurement are the Situation Awareness Rating Technique (SART; Taylor, 1990), Post-assessment of Situation Awareness (Gatsoulis et al., 2010), and the Situational Awareness Rating Scale for self-rating and the Situation Awareness Behavioral Rating Scale (SABARS; Strater et al., 2001) for rating by expert observers.

Experimental methods are the Situation Awareness Global Assessment Technique (SAGAT; Endsley, 1995), the Situation Present Assessment Method (SPAM; Durso et al., 1998), and an eye tracker. It is possible to record an operator when he or she is describing what he or she actually is doing and thinking. Then, what has been recorded can be analyzed (Heikoop et al., 2018). SAGAT was developed for simulation research (Endsley, 2015), and it involves pauses in activity. When the simulation is stopped, the operator is asked about their environmental perception. SPAM was designed for real-time research, so the operator is asked questions about the environment during the activity. SAGAT is more sensitive to experimental differences if the probes into the operator's activities occur at random times rather than saving the questions for the end. SAGAT questions for operators should fulfill several requirements. They should ask only about things that are relevant to the operator's SA; questions must be clear and not be subjective, and it should be easy to decide whether the answer is right or wrong (Endsley, 2019). Eye fixation was found to be a predictor of SAGAT scores (Argyle et al., 2020). SAGAT might face challenges in collecting data about real road traffic because of interruptions.

SA measurement methods based on performance encounter problems with validity because performance and SA might be two different things (Nguyen et al., 2019). Endsley (2020) found that self-rating methods for SA measurements do not correlate with methods such as SAGAT, which aim to objectively measure SA knowledge. The author concluded that these methods measure different things, and self-rating methods should not be considered to measure the general SA. Rather, they should be used to measure only the self-confidence about one's own SA. Combining low SA with high self-confidence might be especially dangerous (Endsley, 2020). Besides, methods based on self-rating after activity have the problem that the subject might have already forgotten details (Nguyen et al., 2019). Experimental objective methods also face some criticism. Salmon et al. (2012) criticized SAGAT as being useless for the analysis of the interaction of different SA components. SAGAT also has been criticized for being unable to capture the unconscious part of SA, for being unsuitable for the study of the SA dynamics (Winter et al., 2019), and for excessive interference with an operator's activity. However, Endsley (2019) did not find such interference when reviewing the research that had examined this problem. The studies she reviewed found no SAGAT negative effects on the operator's performances.

## Situation Awareness in Remotely Controlled Vehicles

With the advancement of RCVs, it is crucial to study the teleoperators SA. Being aware of the environment is pivotal for driving (Zhou et al., 2020). The controls and displays of the RCV should provide accurate information to avoid having the teleoperator stop trusting them. Besides, alarms should warn the teleoperator only when something crucial happens, to avoid displaying false warnings that lead to distrust (Endsley and Jones, 2011). The RCV operators should develop SA in their local environment, i.e., the controls, and displays where they sit, as well as in the remote environment where the vehicle is located, e.g., traffic signs. An important trait is the skills to divide one's attention without losing sight of what is happening elsewhere (Riley et al., 2010). SA in RCVs depends on the interfaces between many vehicle and operational characteristics. Table 1 summarizes driver, environment, and car characteristics that influence a driver's SA. Table 2 summarizes operator, environment, and operation interface characteristics that influence a teleoperator's SA.

Techniques for measuring SA have been developed for many RCVs. Compared to other RCVs, it is more important to study SA for RCCs in real life rather than using simulation because many unexpected things can happen in real traffic especially in countries where traffic rules are less respected (Huang et al., 2006). Endsley and Jones (2011:226–233) summarized challenges for good SA in RCVs, some of which are relevant for RCCs, e.g., (1) the operator's bad sensory information, (2) time delays in the signals transmission, (3) lightning problems at the vehicle's location, and (4) the vehicle's interface complexity and multitasking. A careful design of the RCV's displays and controls can mitigate these problems, thereby allowing for higher SA. The requirements for the RCV's operator could be adapted from the requirements established for ground robots by Endsley and Jones (2011:224–225), i.e., the requirements are that the operator should know the car status (e.g., its speed), its characteristics (e.g., weight), the location of other objects, the status of the motor system, the communication quality, the weather conditions, details of the terrain, and the quality of the car sensors. Besides, the operator must understand how to project all previous experience to deal with present and future situations. In addition, RCC operators should understand the traffic rules in the country, what traffic signs mean, the current status of the traffic lights, car's winkers, whether they are overtaking another vehicle or being overtaken, and the current speed limit.

SA in RCVs often is measured according to the operator's performance. Yanco and Drury (2004) measured the SA of the teleoperator of an urban rescue robot as the number of the collisions with other objects and the time spent adjusting the camera. Velagapudi et al. (2012) measured SA in the context of unmanned aerial vehicles based on the difference between the real positions of colored targets in a video from the vehicle and the positions where the subject thought these targets were on a map. Performance methods also are suitable for studying SA in RCVs. Experts can rate the teleoperator's behavior using SABARS pre-defined scales created for the specific activity, such as those developed by Kaber et al. (2013).

## Suggestions for the Measurement of Situation Awareness in Remotely Controlled Cars

Researchers attempting to measure the SA in RCCs might discover a scarcity of past studies on the SA of RCC-teleoperated drivers. These researchers should follow the following suggestions based on the SA measurement in other environments.

The SA measurement for RCC-teleoperated drivers differs from the measurement of classic car drivers. Teleoperated drivers cannot perceive the car's speed, the slope of the road, or the weight of the load using physical sensation. Besides, they cannot hear the sound of the car's engine telling them about a possible problem. There might be blind spots between cameras, and it might be difficult for the teleoperated drivers to react if there is a loss of connection. The researcher must pay attention to how the operator gets the SA about these things (e.g., repeated checks of the speed indicator due to the lack of physical sensation). SAGAT and SPAM questions concerning these issues might be related to the slope of the road, the quality of the transmission, speed, the position of the wheel, the positions of other objects, and whether all of the car's systems are working correctly. Eye tracker might serve in studying where the operator looks to when searching for information regarding speed, slope, and transmission quality.

Taking the SA of RCCs from the perspective of the system, in addition to the car, other vehicles, pedestrians, and parts of the infrastructure should be considered. Specifically, this means the reaction to viewing a car without a driver. Pedestrians, cyclists, and drivers might behave in different ways in this situation, and this might result in dangerous situations.

All SA measurement methods might be used for studying the SA of RCCs' operators. As with other types of RCVs, performance methods specific for cars might be useful; this includes counting the number of hit skittles or the number of jerks, i.e., episodes of braking too rapidly, measured as occurrences when the second derivative of speed is >−9.9 m/s^3^ (Bagdadi and Várhelyi, 2011). For the analysis of how a driver follows traffic rules, some modification of the Wiener Fahrprobe method (Chaloupka and Risser, 1995) might be useful when one trained researcher rates how the driver/operator follows the traffic rules, and the second researcher watches for unexpected events. This method is a standardized method that has been used for a long time for classic cars. Besides, it assesses drivers'/operators' actual behaviors in a real environment. However, it is not adapted for RCCs, and it requires a significant amount of resources, i.e., the driver should be watched by two researchers, and the observation should be done for a long time in order to see a sufficient number of mistakes. In addition, the method only captures behavior from the perspective of the observer and does not allow to study processes inside the driver's mind.

SAGAT could be useful when studying an operator's behavior on the test tracks. When the monitors are blinded, the control of the car could be taken over by the safety driver. It is more effective than SART (van den Beukel and van der Voort, 2017b). SAGAT disadvantage is that it might cause problems in traffic safety if used in real traffic. It is impossible to use SAGAT there because of the difficulties associated with interruptions.

SPAM might be used when studying the SA of RCC operators in real road traffic. Nevertheless, SAGAT is more successful than SPAM in finding differences between experimental conditions (Endsley, 2019). SPAM possibly could decrease traffic safety in real traffic—it was found to create problems with intrusiveness or workload in 40% of studies reviewed by Endsley (2019). This concern might be decreased by showing “Yes/No” questions on one of the system's displays, as is the case for the Daze system developed by Sirkin et al. (2017). In this case, drivers do not have to listen to SPAM questions. Rather, they can look at the display beside the steering wheel, which requires less attention. However, drivers' interactions with such a display can decrease SA (Wulf et al., 2013), so this solution might be not successful in ensuring traffic safety.

Expert-rating methods like SABARS are important for traffic safety in real road traffic, as they are not interfering with the operators' activities. Given the office-based nature of the remote operation, teleoperated drivers offer better opportunities for observers than the driver in the car. However, SABARS has not been used in the context of car driving or vehicle remote operation.

Self-rating methods are safe in real road traffic. SART was found to be better than SPAM in predicting the performance of remotely controlled robots (Schuster et al., 2012). However, SART has several limitations. It measures a different construct than SAGAT (Endsley, 2020), so it is questionable whether it measures SA, and it is unable to capture the operator's state of SA.

The benefit of eye tracking is its non-intrusiveness, so it does not interfere with traffic safety. Nevertheless, eye tracking does not allow for the assessment of the attention, audio cognition, and information evaluation of the operator's brain (Endsley, 2019). Eye trackers are complicated to use when drivers are inside road vehicles (Nguyen et al., 2019) because of noise (Heikoop et al., 2018). Given the office-based character of the RCC's interface when compared with the driving interface in the car, the eye tracker would provide better data than when used on the road.

The above information is applicable for remotely controlled buses and trucks. The main difference is that trucks and buses are longer and need more cameras. This means that teleoperated drivers should divide attention between more displays at teleoperated driver's interface, which might decrease SA.

## Conclusion

The present study reviews the SA measurement for a teleoperated driver in RCCs. Previous reviews of SA measurement concerned classic car drivers or teleoperators of non-road vehicles; the current text merges both these themes into one review. The study enhances a greater understanding of researchers aiming to decrease the risk of human-factor failure in remote driving. The entire SA measurement methods are available if RCCs are tested on a circuit. Researchers aiming to measure SA on a circuit should prefer objective experimental methods, such as SAGAT. Nevertheless, when studying RCCs on roads in densely populated urban areas, researchers should consider other SA less-intrusive measurement methods and evaluate whether the safety benefits of these methods outbalance their limitations. To date, no intensive research is available on the SA measurements for operators of RCCs, but we expect that it will be studied more extensively in the future.

## Author Contributions

All authors listed have made a substantial, direct and intellectual contribution to the work, and approved it for publication.

## Conflict of Interest

The authors declare that the research was conducted in the absence of any commercial or financial relationships that could be construed as a potential conflict of interest.
